# Quantifying Contributions of Different Factors to Canopy Photosynthesis in 2 Maize Varieties: Development of a Novel 3D Canopy Modeling Pipeline

**DOI:** 10.34133/plantphenomics.0075

**Published:** 2023-07-26

**Authors:** Qingfeng Song, Fusang Liu, Hongyi Bu, Xin-Guang Zhu

**Affiliations:** ^1^ National Key Laboratory of Plant Molecular Genetics, CAS Center for Excellence in Molecular Plant Sciences, Shanghai Institute of Plant Physiology and Ecology, Chinese Academy of Sciences, Shanghai 200032, China.; ^2^ Shanghai Institute of Technical Physics, Chinese Academy of Sciences, Shanghai, China.

## Abstract

Crop yield potential is intrinsically related to canopy photosynthesis; therefore, improving canopy photosynthetic efficiency is a major focus of current efforts to enhance crop yield. Canopy photosynthesis rate (*A_c_*) is influenced by several factors, including plant architecture, leaf chlorophyll content, and leaf photosynthetic properties, which interact with each other. Identifying factors that restrict canopy photosynthesis and target adjustments to improve canopy photosynthesis in a specific crop cultivar pose an important challenge for the breeding community. To address this challenge, we developed a novel pipeline that utilizes factorial analysis, canopy photosynthesis modeling, and phenomics data collected using a 64-camera multi-view stereo system, enabling the dissection of the contributions of different factors to differences in canopy photosynthesis between maize cultivars. We applied this method to 2 maize varieties, W64A and A619, and found that leaf photosynthetic efficiency is the primary determinant (17.5% to 29.2%) of the difference in *A_c_* between 2 maize varieties at all stages, and plant architecture at early stages also contribute to the difference in *A_c_* (5.3% to 6.7%). Additionally, the contributions of each leaf photosynthetic parameter and plant architectural trait were dissected. We also found that the leaf photosynthetic parameters were linearly correlated with *A_c_* and plant architecture traits were non-linearly related to *A_c_*. This study developed a novel pipeline that provides a method for dissecting the relationship among individual phenotypes controlling the complex trait of canopy photosynthesis.

## Graphical abstract

**Figure:**
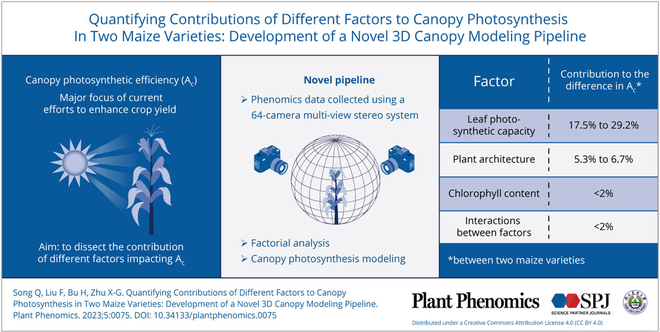

## Introduction

Increasing energy conversion efficiency at the canopy level is regarded as one of the most important options to increase biomass and crop yield potential [1]. Canopy is the aboveground part of plants, and gas exchange measurements indicate that canopy photosynthesis rate is correlated with biomass production and crop yield potential [2–6]. Factors that determine the canopy photosynthesis of crops grown under non-stress conditions include leaf photosynthesis efficiency, canopy architecture, and leaf chlorophyll content [7], which are important targets for breeding programs [8–10]. As these factors interact with each other, canopy photosynthesis is a complex quantitative trait. Experiments under FACE (free air CO_2_ enrichment) have shown that increasing photosynthesis can increase biomass and crop yield potential [11,12]. However, there are contradictory evidences suggesting that leaf photosynthesis might not necessarily be related to crop yield, especially when the canopy architecture and other yield-related traits are changed simultaneously with the changes in leaf photosynthesis [13–15]. Canopy architecture, which determines light interception and distribution in a canopy [16], plays a crucial role in canopy photosynthesis; e.g., erect leaves reduce the shading of lower-layer leaves, and larger leaf area increases the amount of intercepted light by a canopy [17,18]. Leaf chlorophyll content varies greatly among cultivars and affects not only light absorbance but also the photosynthetic efficiency of a leaf [19]. Given these factors and their interactions that control canopy photosynthesis change dynamically, the factor that restricts canopy photosynthesis may shift during the growing season. Accurate identification of the factors controlling photosynthetic efficiency at different stages is essential for efforts to improve photosynthesis and achieve greater yield [20,21].

There are large genetic variations in maize leaf photosynthesis [22]. So far, large-scale phenotyping and genetic studies on leaf photosynthesis have been conducted in rice, poplar, wheat, and other crops [23–25]. However, the study of the genetic mechanism of canopy photosynthesis is limited due to a lack of dissecting methods for canopy photosynthesis, particularly in terms of calculating the contributions of each trait to canopy photosynthesis.

Computational models have been developed to calculate canopy photosynthesis for several crops, such as maize [26,27], wheat [28,29], rice [30–33], soybean [34], sugarcane [35], and sweet pepper [36]*.* These models have aided in the study of various physiological questions, including light use efficiency, ideal plant type, optimal planting densities, and the theoretical creation of virtual super cultivars. We have previously developed a method that uses combinatorial factorial analysis to dissect the contribution of different environmental factors and physiological and architectural traits on canopy photosynthesis for soybean and rice [34,37]. For example, this analysis reveals that at different developmental stages, different features are required to achieve higher photosynthetic efficiency; e.g., at the tillering stage, increasing leaf area index (LAI) is preferred, while at the young panicle differentiation stage, increasing LAI decreases canopy photosynthesis [37].

However, building 3D canopy photosynthesis models for the target plants is time-consuming, and the 3D models developed based on manual [32,33] or semi-automatic image-based measurements [31] still have limited throughput. Canopy models can also be constructed using point cloud, which can be acquired using Light Detection and Ranging (LiDAR), depth camera or time-of-flight camera, or multi-view stereo-derived point clouds based on images captured from multiple views [38–41].

In this study, we developed a novel 3D canopy modeling pipeline consisting of a custom-designed 64-camera multi-view stereo system, ray tracing, and a leaf photosynthetic model for the dissection of major contributions of canopy photosynthesis between cultivars. Specifically, we dissected the contributions of plant architecture, leaf absorbance, and leaf photosynthetic capacity.

## Materials and Methods

### Plant materials

Maize (*Zea mays* L.) inbred lines, A619 and W64A, were cultivated in Songjiang experimental station in Shanghai (30°56' N, 121°8' E) on 2020 July 20. The plants were grown in 21-L pots with four 6-mm-diameter holes at the bottom to facilitate the drainage of excess water. The bottom layer of the pots was filled with 1 l of ceramsite, followed by 20 l of mixed soil (garden soil:peat soil:vermiculite = 5:3:2 by volume). Prior to sowing, 20 g of urea and 20 g of N-P-K compound fertilizer (a total of 12.2 g of N, 3 g of P_2_O_5_, and 3 g of K_2_O per pot) were applied. The pots were placed in direct sunlight and irrigation was performed as necessary during the growing season.

### The new pipeline of 3D canopy modeling

**Fig. 1.**
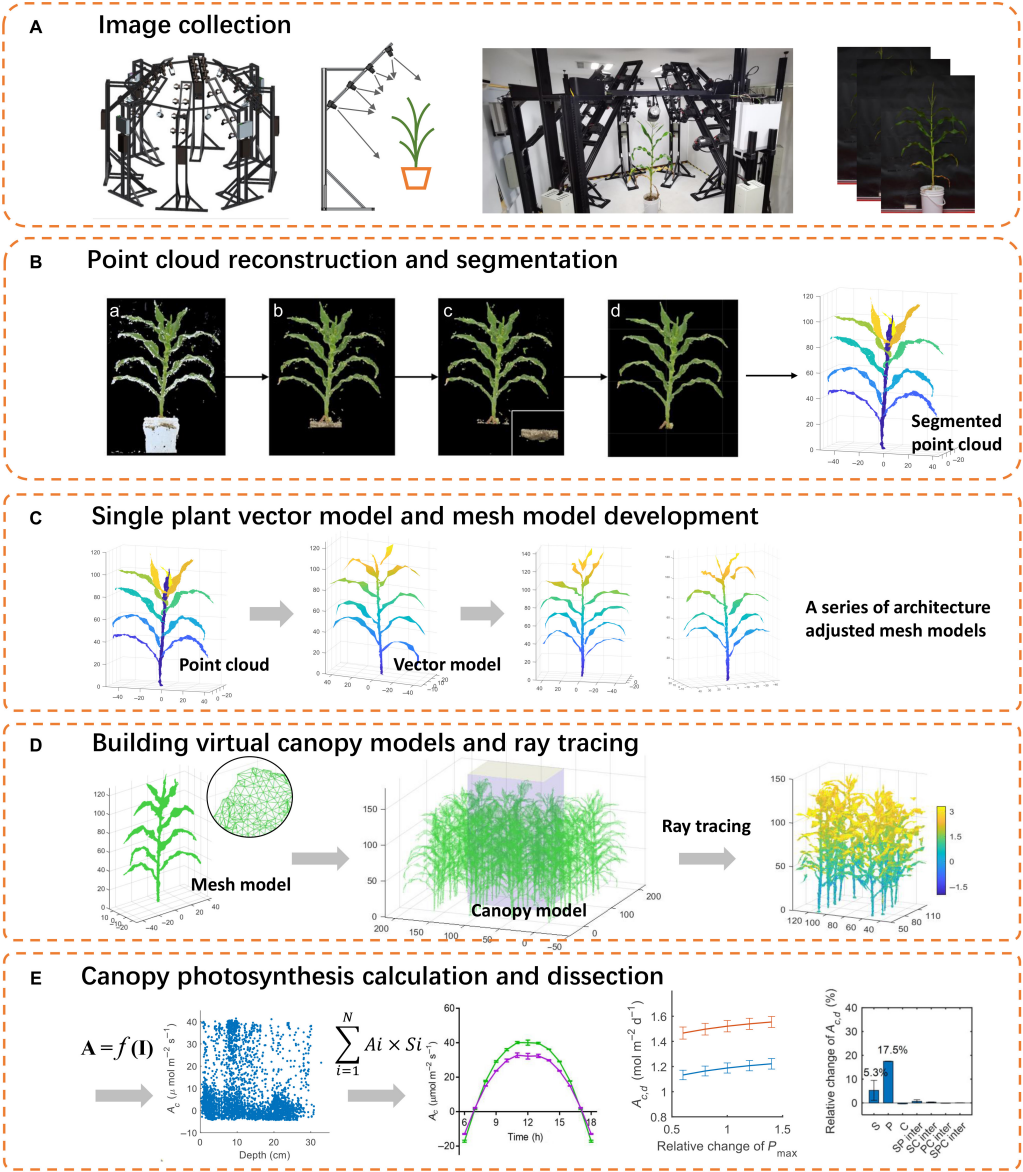
The pipeline of 3D canopy modeling. The pipeline includes several steps, including (A) image collection with an MVS64 system, (B) point cloud reconstruction and segmentation, (C) a single plant vector model was built based on the segmented point cloud and mesh models of virtual plants were developed, (D) building canopy models and ray tracing, and (E) canopy photosynthesis calculation, sensitivity analysis, and factorial dissection.

The pipeline includes several steps, including (1) image collection, (2) point cloud reconstruction and segmentation, (3) single plant vector model and mesh model development, (4) building virtual canopy models and ray tracing, and (5) canopy photosynthesis calculation and dissection (Fig. 1).

Step 1. In this study, we used a custom-designed multi-view stereo system, MVS64, to collect images of plant grown in pots.

Step 2. Point cloud was reconstructed with software Agisoft Metashape with an input of the 64 images collected from MVS64. Point cloud denoising and segmentation was performed to separate leaves and stem. All leaves were numbered by 1, 2, 3, etc. Those leaves that were lower than the center of the plant were defined as lower layer leaves and were labeled with 1; those leaves that were higher than the center of the plant were defined as up layer leaves and were labeled with 2. The maize single plant segmented point cloud was built.

Step 3. Based on the segmented point cloud data, a vector model was constructed. The leaves in the vector model were presented as vectors, in which it is easy to adjust the leaf length, leaf width, leaf angle, etc. Using the vector model, a series of virtual plant mesh models were generated.

Step 4. A canopy model was built based on the single plant mesh model, and ray tracing simulation was performed with the FastTracer software.

Step 5. A leaf photosynthetic light response curve was used to calculate the photosynthesis of every leaf triangle facet, and the canopy photosynthesis was calculated. Sensitivity analysis and factorial dissection analysis were carried out.

More details of the above steps are shown as follows.

### Development of a 64-camera multi-view stereo system

A new phenotyping platform (Fig. 1A and a video in the Supplementary Materials) was developed for instantaneous phenotyping of plant architectures for the construction of canopy models. The platform includes 64 cameras (EOS 1300D DSLR, Canon, Inc., Tokyo, Japan), 8 terminal controllers, 4 computer nodes, and one host computer. All the cameras, terminal controllers and computer nodes are installed on a mechanical skeleton (Fig. 1A). There is a cross marker on the ground showing the center of the platform. The mechanical skeleton is fixed on the floor and ceiling of the room for stabilization. Four flash lamps are used during photographing. To obtain a uniform and stable light environment, the platform is surrounded by curtains and the floor is covered by material that scatters light. A maize plant grown in a pot was moved to the center of the platform and all the 64 cameras took images simultaneously. The cameras are controlled by the host computer. The host computer connects with 4 nodes of computers, each of which links 2 terminal controllers. One terminal controller links 8 cameras, which are located at 4 different heights. The phenotyping platform is operated with software installed on the host computer.

### Point cloud calculation and pre-processing

Images from the phenotyping platform are used to generate dense point clouds by the software Agisoft Metashape Professional Edition (Agisoft LLC, St. Petersburg, Russia; version 1.6.1). The point clouds data include the information of *X*–*Y*–*Z* coordinates, color (RGB), and normal vector (of the object surface, such as a leaf or a stem) of the points. Noise points were removed with a series of processes with the point cloud toolkit in MATLAB (R2020b, MathWorks, USA). The soil plane is extracted by the function *pcfitplane* with the parameter of allowable distance 5 cm. Point clouds are further denoised with the *pcsegdist* (threshold of Euclidean distance 5 mm) and *pcdenoise* function (threshold of standard deviation 0.3 away from the averaged distance of between each point and its nearest 50 neighbors). To verify the accuracy of 3D point clouds acquired from the multi-view stereo system, we used LiDAR (FARO S70 series) with an accuracy of 1 mm to scan plants for point clouds.

### Plant organ segmentation and architectural parameter extraction

The method presented by Liu et al. [42] for plant organ segmentation is employed to segment each leaf from the stem. The method is a combination of a skeleton extraction algorithm and a region growing algorithm, which has been proven to be effective for maize by Liu et al. [43]. The algorithm consists of 3 major parts, (a) extracting the leaf skeleton, (b) classifying the point cloud into clusters, and (c) merging unknown clusters. Once all leaves are separated from the stem, the architectural traits of the leaves are extracted using the method described [42]. The extracted leaf architectural traits include leaf length, leaf width, leaf base height, and leaf area. The leaf area was calculated as the summation of mesh area of a leaf. The calculated leaf area was compared with the leaf area determined using an empirical model [44], which takes into account leaf length and width. To evaluate the extracted traits, *R*^2^ and RMSE are calculated based on measured data.

### Development of a 3D vector model for leaves based on point cloud data

To adjust length, width, angle, and curvature of the leaves, we developed a 3D vector model based on the point cloud. First, the Dijkstra algorithm was used to calculate the main path of a leaf from the leaf base to tip. Next, a series of planes were selected to cut the leaf into sections in order to detect its edges. Finally, vectors representing the length of the leaf were defined using the points along the main path from the leaf base to the tip, and vectors representing the width of the leaf were defined using the points between the leaf's central vein and its edges. We multiplied the vectors representing leaf length by a factor to adjust its length. Similarly, to adjust the leaf width, we multiplied the vectors representing leaf width by a factor.

### Building 3D canopy model based on point cloud data

Point clouds of a single plant are converted into mesh models and used for constructing virtual canopies (Fig. 1C). The Crust method [45] was used for triangulation and then abnormal facets (or triangles) are filtered with a statistical method used in a previous study [43]. Each canopy model is built with 4 different individual plants, and the data of the 4 plants are used repeatedly. One canopy model includes 4 rows with a 55-cm distance between rows and 13 plants per row with a 15-cm distance between plants.

### Simulating light distribution with ray tracing algorithm

The light distribution in the canopy model is simulated with ray tracing algorithm using the software *FastTracer* [32]. In the model, the center area of 110 cm × 75 cm (2 rows × 5 plants/row) is used for ray tracing simulation and canopy photosynthesis calculation to avoid boundary effect (Fig. 1D). The meta information including date and location are used as input to the software. In a ray tracing algorithm for a particular canopy, the leaf reflectance (*R*) and transmittance (*T*) need to be parameterized. The parameterization is based on the relationships between the SPAD values measured with SPAD502Plus (Konica Minolta, Japan), which represents leaf chlorophyll concentration [46], and *R* and *T*. The *R* and *T* values are calculated for photosynthetic active radiation (PAR) of solar light with Eqs. 1 and 2 [47]. In these equations, the *R_i_*, *T_i_*, and *I_i_* are reflectance, transmittance, and incident light, respectively, at wavelength *i*. A spectrometer and an integrating sphere (Ocean Optics, Dunedin, FL, USA) are used for the measurement of *R_i_* and *T_i_*.

$$R=\frac{\Sigma_{i=400}^{700}R_{i}*I_{i}}{\Sigma_{i=400}^{700}I_{i}}$$ (1)

$$T=\frac{\Sigma_{i=400}^{700}T_{i}*I_{i}}{\Sigma_{i=400}^{700}I_{i}}$$ (2)

The incident light is predicted with a climate model and the direct PPFD and diffuse PPFD are predicted based on an atmosphere transmittance of 0.7.

### Calculation of canopy photosynthesis rate

The classic non-rectangular hyperbola leaf photosynthesis model (Eq. 3) [48] is used to calculate the photosynthetic CO_2_ assimilation rate for every triangle of individual leaves in a canopy model according to the absorbed PAR by each triangle (Fig. 1E). *A* is the leaf photosynthesis rate and *I* is the incident photosynthetic photon flux density. *P*_max_ is the leaf photosynthetic CO_2_ assimilation rate under saturated light. *ϕ* is the quantum yield of CO_2_ assimilation. *θ* is the curve convexity, which describes the sharpness of the transition in the light response curve. The leaf photosynthesis model (Eq. 3) was parameterized by fitting the light response curves of photosynthesis measured with a leaf gas exchange system LI-6400XT (LI-COR, Lincoln, NE, USA). The fitting results are shown in Table S1. The top and bottom layer leaves were measured separately, and 2 models were used to represent them because of the physiological differences between the top and bottom layer’s leaves. The measurements were done under a reference CO_2_ concentration of 400 μmol mol^−1^ and photosynthetic photon flux density (PPFD) changing from high light to low light with 2-min intervals (2,000, 1,500, 1,000, 800, 600, 400, 300, 200, 150, 100, 50, and 0 μmol·m^−2^·s^−1^).

$$A=\frac{\phi I+P_{\max}-\sqrt{{\left(\phi I+P_{\max}\right)}^{2}}-4\theta \phi {\mathrm{IP}}_{\max}}{2\theta }-R_{d}$$ (3)

The canopy photosynthesis rate equals the sum of all leaf photosynthesis rates multiplied by leaf area. The diurnal canopy photosynthesis rate is calculated based on the simulated light environments on an hourly interval (Fig. 1E).

### Dissection of factors controlling canopy photosynthesis

The algorithm of dissection analysis follows Refs. [34,37]. The difference of canopy photosynthetic CO_2_ uptake rate (*A_c_*) between 2 lines (or 2 cultivars) can be attributed to 3 different traits, e.g., canopy structure, chlorophyll content, and leaf photosynthesis (Eqs. 4 to 10). The *A_c_*(*X*) represents the *A_c_* with trait *X* from A619 and the others from W64A, e.g., *A_c_*(S) is the *A_c_* with trait S (canopy architecture) from A619 and the other traits from W64A. The symbol O represents the control canopy model, the symbol S represents the canopy model with replaced 3D canopy structure, P represents the canopy model with replaced leaf photosynthesis, C represents the canopy model with replaced leaf chlorophyll contents. *c*(*X*) represents the contribution of trait *X*. The difference of *A_c_*(S) and *A_c_*(O), (*A_c_*(S) − *A_c_*(O)), is the contribution of the 3D canopy model to the canopy photosynthesis. Similarly, *A_c_*(S, P) represents the canopy photosynthesis rate of the canopy model with trait S and P from A619 and the other traits from W64A; *A_c_*(S, P, C) represents canopy model with all the trait S, P, and C from A619.

$$A_{c}(S)-A_{c}(O)=c\;(S)\;$$ (4)

$$A_{c}(P)-A_{c}(O)=c\;(P)$$ (5)

$$A_{c}(C)-A_{c}(O)=c\;(C)$$ (6)

$$A_{c}(S,P)-A_{c}(O)=c\;(S)+c\;(P)+c\;(\mathrm{SP})$$ (7)

$$A_{c}(S,C)-A_{c}(O)=c\;(S)+c\;(C)+c\;(\mathrm{SC})$$ (8)

$$A_{c}(P,C)-A_{c}(O)=c\;(P)+c\;(C)+c\;(\mathrm{PC})$$ (9)

$$\begin{array}{c} A_{c}(S,P,C)-A_{c}(O)=c\;(S)+c\;(P)+c\;(C)+\\ c\;(\mathrm{SP})+c\;(\mathrm{SC})+c\;(\mathrm{PC})+c\;(\mathrm{SPC}) \end{array}$$ (10)

The 3D structural parameters, leaf photosynthesis, and leaf SPAD values of the 2 inbred lines, e.g., W64A and A619, were measured. With these data, we built a series of virtual canopy models using different combinations of the 3 traits from the 2 inbred lines. All the *A_c_*(S), *A_c_*(P), *A_c_*(C), *A_c_*(S, P), *A_c_*(S, C), *A_c_*(P, C), *A_c_*(S, P, C), and *A_c_*(O) can be calculated with canopy models. Then, the contributions of individual traits and interactions between 2 traits or among 3 traits are derived from the following equations (Eqs. 11 to 17).

$$c\;(S)=A_{c}(S)-A_{c}(O)$$ (11)

$$c\;(P)=A_{c}(P)-A_{c}(O)$$ (12)

$$c\;(C)=A_{c}(C)-A_{c}(O)$$ (13)

$$c\;(C)=A_{c}(C)-A_{c}(O)$$ (14)

$$c\;(\text{SC})=A_{c}(S,C)-A_{c}(S)-A_{c}(C)+A_{c}(O)$$ (15)

$$c\;(\text{SC})=A_{c}(S,C)-A_{c}(S)-A_{c}(C)+A_{c}(O)$$ (16)

$$\begin{array}{c} c\;(\mathrm{SPC})=A_{c}(S,P,C)-A_{c}(O)+A_{c}(S)+\\ A_{c}(P)+A_{c}(C)-A_{c}(S,P)-A_{c}(S)C)-A_{c}(P,C) \end{array}$$ (17)

To better understand the contribution of these individual features and their interactions to canopy photosynthetic CO_2_ uptake rate, we calculated these contributions (e.g., c(S), c(P)) to the canopy photosynthetic CO_2_ uptake rate of W64A at each stage, respectively. The contributions are converted from their absolute values to relative values representing the proportion of *A_c_* increase by replacing features of W64A by features from A619.

## Results

### The accurate assessment of point cloud data from MVS64 using LiDAR and measured plant architectural data

**Fig. 2.**
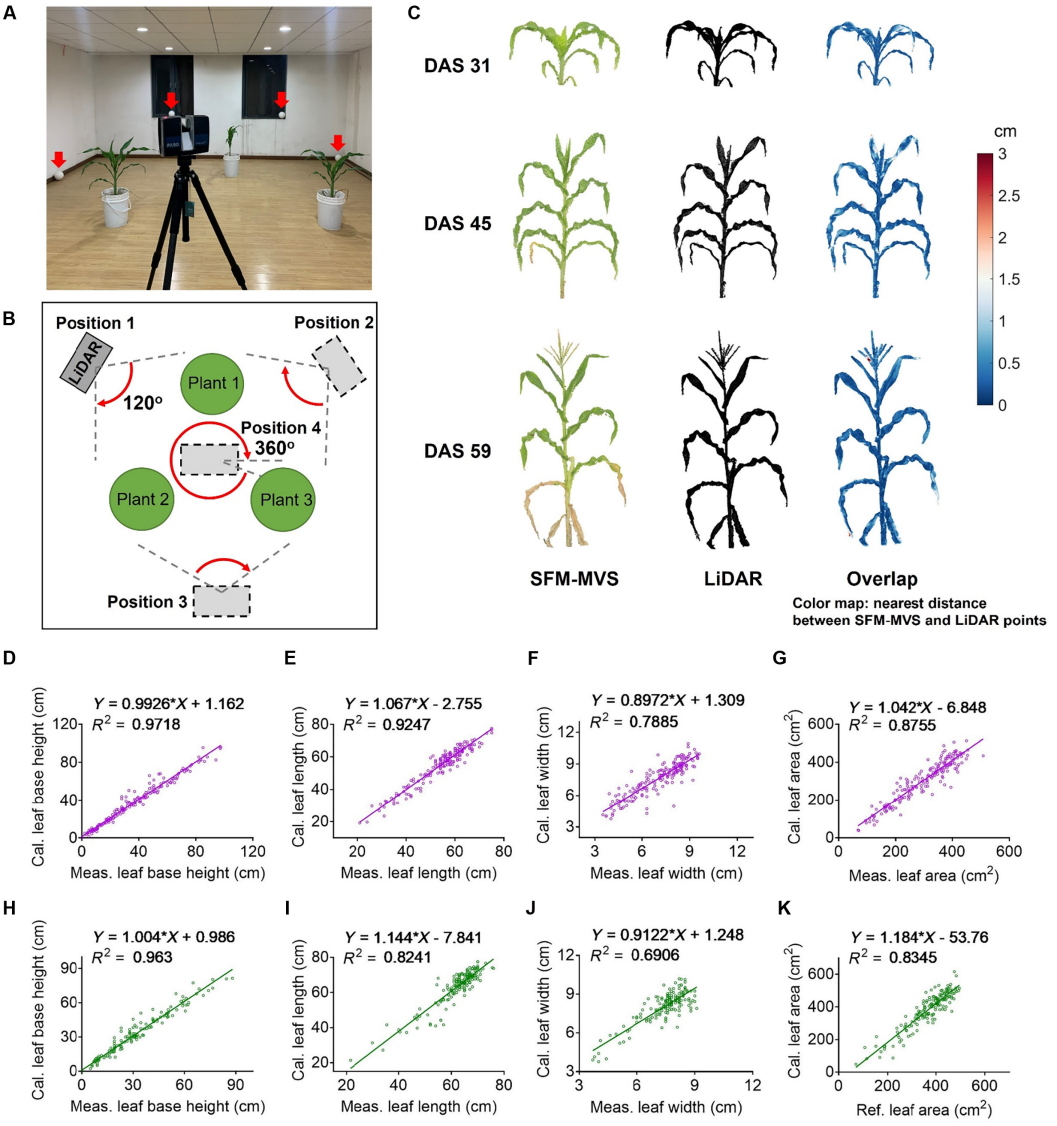
Validation of point cloud accuracy and extracted parameters with LiDAR and manually measured data. (A and B) The setup of the LiDAR device and a diagram showing the method of scanning multiple plants simultaneously. The LiDAR device was moved between different positions, and at positions 1, 2, and 3, the LiDAR scanned from 0° to 120°; at position 4, the LiDAR scanned from 0° to 360°. (C) Alignment between LiDAR point cloud and SFM-MVS point cloud. The point cloud of SFM-MVS includes R-G-B color information while the point cloud of LiDAR has no color information. The color of the overlap figures shows distance between 2 point clouds. (D to K) The correlation between calculated plant architectural traits and measured data. These traits include leaf base height (D and H), leaf length (E and I), and leaf width (F and J). The correlation between calculated leaf area from point cloud and reference leaf area calculated with commercial software (G and K). Data in (D) to (G) are from line W64A and those in (H) to (K) are from line A619.

The study aimed to construct a 3D canopy structure model by the point clouds for individual plants of 2 inbred lines, W64A and A619, at 5 stages: 31st, 38th, 45th, 52nd, and 59th days after sowing (DAS). The point clouds were computed with the structure-from-motion and multi-view stereo (SFM-MVS) method (Fig. 1A). The images were taken by an MVS system composed of 64 cameras (MVS64) simultaneously from 64 views (Fig. 1A). We evaluated the accuracy of point cloud using LiDAR on the same day that the images were taken, following the method of previous studies [40,41] (Fig. 2A and B). The point cloud acquired from the MVS64 was matched to the point cloud obtained from LiDAR and the median distance between the 2 clouds is 3 mm (Fig. 2C). This comparison demonstrated that the SFM-MVS method accurately obtained the point cloud and can be utilized for extracting plant architectural parameters.

Furthermore, we compared the extracted plant architectural traits (i.e., leaf base height, leaf length, leaf width, and leaf area) with the measured data (Fig. 2D to K). The extracted leaf base height displayed substantial correlation with the measured data (*R*^2^ = 0.972, RMSE = 4.262 for W64A and *R*^2^ = 0.963, RMSE = 4.114 for A619). The extracted leaf length is correlated with the measured leaf length (*R*^2^ = 0.925, RMSE = 3.232 for W64A and *R*^2^ = 0.824, RMSE = 4.297 for A619). The RMSE for leaf width was RMSE = 0.742 cm for W64A and RMSE = 0.666 cm for A619, indicating the high accuracy of leaf width extraction from the point cloud. Nonetheless, the correlation coefficient (*R*^2^) between the extracted and measured leaf width (*R*^2^ = 0.789 for W64A, *R*^2^ = 0.691 for A619) was not as high as it was for leaf length and leaf base height. The leaf area was estimated from the measured leaf length and leaf width. The correlations between extracted and measured leaf area were moderate (*R*^2^ = 0.876, RMSE = 38.442 for W64A and *R*^2^ = 0.835, RMSE = 44.888 for A619) (Fig. 2D to K). Table 1 presents the results of linear fitting.

**Table 1.** The parameters and goodness of linear fitting between the extracted plant architectural parameters from point cloud and the manually measured plant architectural parameters. The plant architectural parameters include leaf base height, leaf length, leaf width, and leaf area. *N* is the number of leaves used for the linear fitting and the data are from 5 stages and 8 plants for each stage. The fitting equation *Y* = *p*1 * *X* + *p*2, where *X* is measured data and *Y* is calculated results. *R*^2^ and root mean squared error (RMSE) of the linear fitting are presented.

Lines	Architectural traits	*p*1 (with 95% confidence bounds)	*p*2 (with 95% confidence bounds)	*R* ^2^	RMSE	*N*
W64A	Leaf base height	0.993 (0.968, 1.017)	1.162 (0.140, 2.183)	0.972	4.262	184
	Leaf length	1.067 (1.022, 1.111)	−2.755 (−5.218, −0.292)	0.925	3.232	184
	Leaf width	0.897 (0.829, 0.965)	1.309 (0.819, 1.798)	0.789	0.742	184
	Leaf area	1.042 (0.985, 1.100)	−6.848 (−24.890, 11.190)	0.876	38.442	184
A619	Leaf base height	1.004 (0.974, 1.035)	0.986 (−0.076, 2.048)	0.963	4.114	163
	Leaf length	1.144 (1.061, 1.226)	−7.841 (−12.990, −2.691)	0.824	4.297	163
	Leaf width	0.912 (0.817, 1.007)	1.248 (0.532, 1.965)	0.691	0.666	163
	Leaf area	1.184 (1.102, 1.267)	−53.76 (−84.84, −22.67)	0.835	44.888	163

### Variation of plant architecture, chlorophyll content, and leaf photosynthetic efficiency between 2 maize inbred lines at 5 stages

**Fig. 3.**
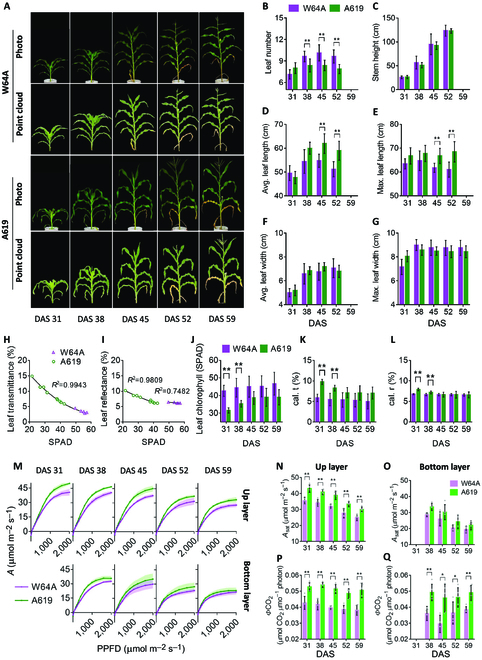
The phenotype data of plant architecture, leaf reflectance and transmittance, leaf chlorophyll, and leaf photosynthesis. (A) Photos and point clouds of 2 maize inbred lines with different architecture, lines W64A and A619, at 5 developmental stages including the 31st, 38th, 45th, 52nd, and 59th day after sowing (DAS). (B to G) Plant architectural parameters, including leaf number per plant, stem height, average leaf length, maximal leaf length, average leaf width, and maximal leaf width. These parameters were measured at 4 stages, and the architecture at the 59th DAS was assumed to be the same as the 52nd DAS. (H and I) The relationship between leaf transmittance and SPAD, and the relationship between leaf reflectance and SPAD. (J) Leaf chlorophyll contents (SPAD values) for 2 maize inbred lines (W64A and A619) at different developmental stages (31st, 38th, 45th, 52nd, and 59th days after sowing, DAS). (K and L) Calculated leaf transmittance and reflectance for the 2 maize inbred lines at 5 stages. Data in (B) to (G) and (J) to (L) are shown as mean ± SD (*n* = 8 biological replicates). (M) Light response curve of photosynthesis for up layer and bottom layer leaves of lines W64A and A619. Data are shown as mean ± SD (*n* = 5 biological replicates) and the *P* values of statistical analysis are shown in Table S2. (N and O) Leaf net photosynthetic CO_2_ assimilation rate (*A*_sat_) measured under saturated light (photosynthetic photon flux density, PPFD, 1,200 μmol m^−2^ s^−1^) for the up layer and bottom layer of the lines W64A and A619 at 5 stages. (P and Q) Quantum yield of CO_2_ assimilation (ΦCO_2_), the initial slope of the light response curve of leaf photosynthesis, for the up layer and bottom layer of the lines W64A and A619 at 5 stages. The slope was fitted with data points measured under PPFD 50, 100, 150, and 200 μmol m^−2^ s^−1^. Data in (N) to (Q) are shown as mean ± SD (*n* = 5 biological replicates), ** represent *P* < 0.01 and * represent *P* < 0.05 determined by the Student’s *t* test.

To understand the contributions of different traits to the canopy photosynthesis for the 2 maize lines W64A and A619, we measured the major traits influencing canopy photosynthesis, i.e., plant architecture, leaf optics, and leaf photosynthesis. Firstly, the plant architecture was dramatically different between the 2 lines (Fig. 3A). The 3D point clouds were acquired (Fig. 3A) with the structure from motion (SFM) approach with the new phenotyping platform developed in this study (Fig. 1A and B). We also measure the plant architectural traits manually. Leaf number of W64A was not significantly different from A619 at the first stage (31 DAS), but significantly higher than A619 at the other stages (*P* < 0.01 for 38, 45, and 52 DAS) (Fig. 3B). The stem height of the 2 inbred lines was not significantly different (Fig. 3C). The average and maximal leaf length of A619 was significantly longer than W64A at mature stages (*P* < 0.01 for 45 and 52 DAS) (Fig. 3D and E). There was no significant difference in leaf width between the 2 lines (Fig. 3F and G).

Leaf chlorophyll content of all the leaves in one plant was measured with a chlorophyll meter, SPAD, and the averaged SPAD values of all leaves of A619 are significantly lower than W64A at the first 2 stages (*P* < 0.01 for 31 and 38 DAS) (Fig. 3J). To calculate the leaf reflectance and transmittance for parameterizing ray tracing algorithm, we measured the relationship between SPAD with reflectance and transmittance. The relationship between leaf transmittance (*t*) and SPAD for A619 and W64A together was fitted with a quadratic model (Eq. 1, *R*^2^ = 0.9943) (Fig. 3H). The relationship between leaf reflectance (*r*_A619_ and *r*_W64A_) and SPAD was fitted with a quadratic model of A619 (Eq. 2, *R*^2^ = 0.9809) and a linear model for W64A (Eq. 3, *R*^2^ = 0.7482) (Fig. 3I).

$$t\;=\;0.006319\;*\;{\text{SPAD}}^{2}\;-\;0.8241\;*\;\text{SPAD}\;+\;29.49$$ (18)

$$r_{A619}=0.002478\;*\;{\text{SPAD}}^{2}-0.3554\;*\;\text{SPAD}\;+\;16.66$$ (19)

$$r_{W64A}=-0.04863\;*\;\text{SPAD}\;+\;8.856$$ (20)

With these relationships, the transmittance and reflectance of each leaf were calculated. The averaged leaf transmittance and reflectance of A619 were significantly higher than those of W64A at 31 and 38 DAS, but not significantly different at 45, 52, and 59 DAS (Fig. 3K and L).

The light response curves of the leaf photosynthesis rate show that, for up layer leaves, A619 had a higher leaf photosynthetic rate (*A*) than W64A under most light levels (*P* < 0.05) (Fig. 3M). The *P* values of statistical analysis are shown in Table S2. For bottom layer leaves, *A* of A619 was higher than W64A at the 38th DAS under most light levels (*P* < 0.05) (Table S2), but not significantly different at the other stages (45, 52, and 59 DAS) under most light levels (Table S2). Furthermore, we compared the leaf photosynthetic CO_2_ assimilation rate (*A*_sat_) under saturated light conditions (PPFD = 1,200 μmol m^−2^ s^−1^). The *A*_sat_ of A619 was significantly higher than that of W64A for up layer leaves at all 5 stages (Fig. 3N), while the difference of *A*_sat_ for bottom layer leaves was not significant (Fig. 3O). The quantum yield of CO_2_ assimilation (Φ_CO2_), which is the initial slope of the light response curve, was quantified by linear fitting the data measured under PPFD below 200 μmol m^−2^ s^−1^. The *Φ*_CO2_ of A619 was significantly higher than that of W64A for both up and bottom layers at all 5 stages (Fig. 3P and Q).

Although the difference of these traits between the 2 maize lines can be measured, their contribution to canopy photosynthesis is still not known and which trait is the major factor controlling the difference of canopy photosynthesis between the 2 lines is not clear.

### Influences of photosynthetic and architectural traits on canopy photosynthesis in 2 maize varieties explored using 3D canopy models

**Fig. 4.**
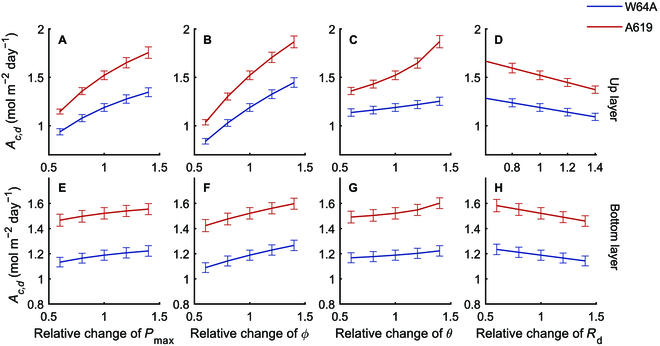
Relationship between leaf photosynthetic parameters (up and bottom layer leaves) and canopy photosynthesis rate (*A_c_*) at the second stage (DAS 38) for the 2 maize inbred lines (blue line: W64A; red line: A619) derived with the modeling pipeline. Leaf photosynthetic parameters include the maximal photosynthesis rate *P*_max_, quantum yield *ϕ*, convexity of light curve *θ*, and respiration rate *R*_d_ of up layer (A to D) and bottom layer leaves (E to H). Data used for building models were measured data on the 38th day after sowing. Data were shown as mean ± SD (*n* = 5 repeats of model simulation). Simulations for other stages are shown in Figs. S1 to S4.

We constructed 3D canopy models for 2 maize varieties using the data obtained to investigate how the traits measured above influence canopy photosynthesis. Using the models, we proportionally adjusted each parameter of the leaf photosynthetic light response curve (*P*_max_, *ϕ*,·*θ*, and *R*_d_) by a range of 0.6 to 1.4 to determine their effect on daily total canopy photosynthetic CO_2_ uptake (*A_c,d_*). Simulation results show that the *A_c_* was almost linearly correlated with *P*_max_*, ϕ, θ*, and *R*_d_ (Fig. 4)*,* except for the *θ* of A619 (Fig. 4C and G). We performed the simulations at 5 stages and the results were consistent (Figs. S1 to S4). The slopes of these curves were higher for the upper layer (Fig. 4A to D) than for the bottom layer (Fig. 4E to H), indicating a greater impact of the parameters of the upper layer on *A_c_* due to higher leaf area and absorbed light than the bottom layer.

**Fig. 5.**
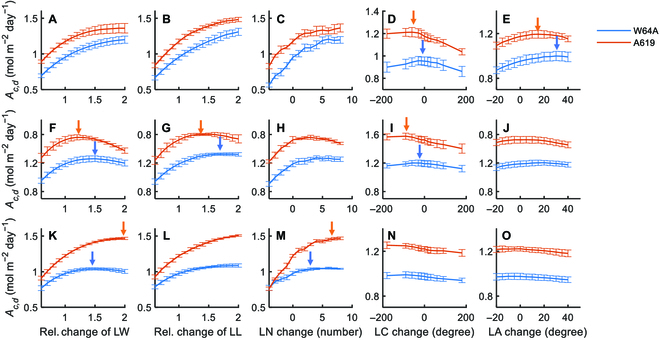
Non-linear relationship between plant architectural traits and daily canopy photosynthesis (*A_c_*) calculated with the modeling pipeline. The sensitivity analysis was done at different stages: DAS 31 (A to E), DAS 38 (F to J), and DAS 59 (K to O) for the 2 maize W64A (blue line) and A619 (red line). Plant architectural parameters are LW: leaf width; LL: leaf length; LN: leaf number; LC: leaf curvature; and LA: leaf angle. Arrows show the optimal values of adjustment. The relative change of traits (for LW and LL) was the multiplied factor based on the original value of cultivar W64A and A619. The LN change number was the leaf number increased or decreased. The LC and LA change degree was the degree increased or decreased based on the original plant. Data were shown as mean ± SD (*n* = 5 repeats of model simulation). Simulations for the other 2 stages (DAS 45 and 52) are shown in Fig. S5.

Next, we evaluated how the plant architectural parameters affect canopy photosynthesis (*A_c_*) for the 2 maize varieties. Using the above models, we generated virtual canopies and proportional manipulated leaf width (LW) and leaf length (LL) (ranging from 0.6 to 2.0), additive changed leaf number (LN) (from −4 to 8), leaf curvature (LC) (from −180° to 180°) and leaf angle (LA) (from −20° to 40°) to determine the daily total canopy photosynthetic CO_2_ uptake (*A_c,d_*). The relationships between these architectural traits and *A_c,d_* were non-linear in most situations (Fig. 5). The optimal values of some traits can be identified from the simulation, such as leaf curvature and leaf angle at DAS 31 (Fig. 5D and E) and leaf width and length at DAS 38 (Fig. 5G and H).

**Fig. 6.**
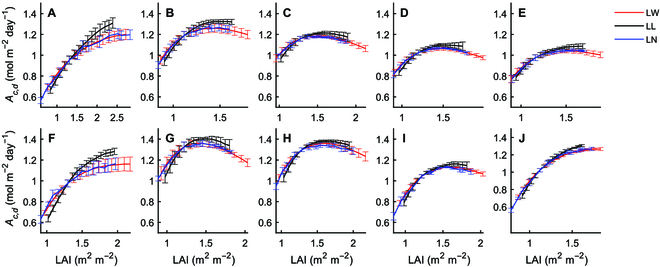
The shared function between leaf width, leaf length, and leaf number in controlling *A_c,d_* shown by the relationship between diurnal canopy photosynthetic CO_2_ uptake rate (*A_c,d_*) and leaf area index (LAI). The analysis was performed for W64A (A to E) and A619 (F to J) with the modeling pipeline. The LAI variation in the horizontal axis was achieved by changing leaf width (red line), leaf length (black line), and leaf number (blue line), respectively. Data are shown as mean ± SD (*n* = 5 repeats of model simulation).

**Fig. 7.**
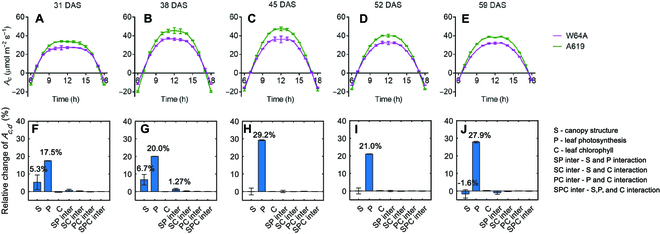
Difference of diurnal canopy photosynthesis between the 2 main inbred lines (A to E) and the dissected contributions of canopy structure, leaf photosynthesis, leaf chlorophyll, and their interactions (F to J). Two inbred lines: W64A (purple line) and A619 (green line) at 5 stages: 31, 38, 45, 52, and 59 days after sowing (DAS). Data were calculated with the modeling pipeline and shown as mean ± SD (*n* = 5 times of model calculation).

Leaf width, leaf length, and leaf number were major traits determining LAI (the ratio of leaf area over ground area). We studied how the 3 traits differ in determining LAI affect canopy photosynthesis by analyzing the relationship between LAI and *A_c,d_* when individually changing the traits. The simulation portrayed that *A_c,d_* had a more significant increase when altering leaf length than when manipulating leaf width or leaf number (Fig. 7). In addition, the impact on *A_c,d_* was similar when modifying either leaf width or leaf number. The results for cultivar A619 were consistent with W64A. The optimal values for the adjustment varied across the 2 maize varieties and developmental stages as indicated by Fig. 6 and Fig. S5, indicating the influence of one architectural trait on other architectural and photosynthetic attributes.

### Contributions of canopy structure, leaf light absorbance, and leaf photosynthesis to canopy photosynthesis dissected with model

The diurnal canopy photosynthetic CO_2_ uptake rate (*A_c_*) was calculated for the 2 inbred lines at 5 stages: DAS 31, 38, 45, 52, and 59. Results indicate that the A619 had a significantly higher *A_c_* than W64A at all stages (Fig. 7A to E). To determine the factors contributing to the difference in canopy photosynthesis between W64A and A619, virtual canopies were created with different combinations of traits from the 2 maize inbred lines (Table 2). The whole day *A_c_* (*A_c,d_*) of these canopies was then calculated. We used equations (Eqs. 11 to 17) to evaluate the contributions of each trait and the interactions between them. Analysis presented in Fig. 7F to J shows that leaf photosynthesis had the most significant impact on *A_c,d_*. The trait of leaf photosynthesis from A619 increased *A_c,d_* of W64A by 17.5% to 29.2% at different stages, while the trait of canopy structure from A619 increased *A_c,d_* of W64A by −1.6% to 6.7% at different stages (Fig. 7F to J). The contributions of trait related to leaf transmittance and reflectance (predicted with chlorophyll content) and the interactions between 2 traits or among 3 traits to *A_c,d_* was less than 2% (Fig. 7F to J).

**Table 2.** Scenarios used to calculate net canopy photosynthetic CO_2_ uptake rate (*A*_*c*_), which is used to dissect the contributions of individual trait (plant architecture, leaf photosynthesis, and chlorophyll content) and their interactions to the difference in *A*_*c*_. Symbol shows different combinations; “W” means the trait is from the inbred line W64A and “A” means the trait is from the inbred line A619.

Symbol	Plant architecture	Leaf photosynthesis	Chlorophyll content
O	W	W	W
S	A	W	W
P	W	A	W
C	W	W	A
S,C	A	W	A
S,P	A	A	W
P,C	W	A	A
S,P,C	A	A	A

**Fig. 8.**
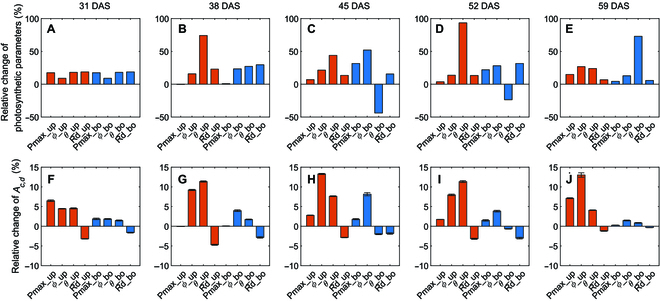
The relative changes of leaf photosynthetic traits (*P*_max_, *ϕ*, and *θ*) of up layer (red bars) and bottom layer (blue bars) (A to E) and their contributions to the difference of canopy photosynthesis between the 2 varieties (F to J). Data were calculated with the modeling pipeline and shown as mean ± SD (*n* = 5 times of model calculation).

**Fig. 9.**
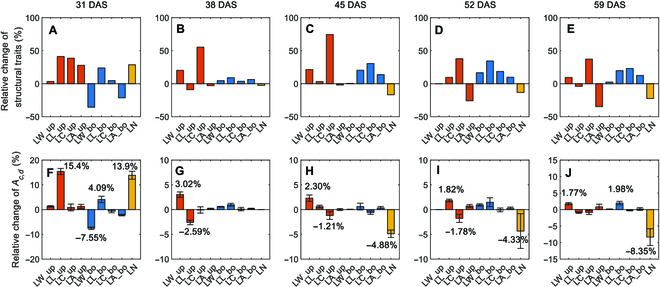
The relative changes of canopy architectural traits (LW: leaf width; LL: leaf length; LC: leaf curvature; LA: leaf angle) of up layer (red bars) and bottom layer (blue bars) as well as the leaf number (LN) (yellow bars) (A to E) and their contributions to the difference of daily canopy photosynthesis (*A_c,d_*) between the 2 varieties (F to J). Data were calculated with the modeling pipeline and shown as mean ± SD (*n* = 5 times of model calculation).

To understand the contributions of specific traits to *A_c,d_*. We divided the leaf photosynthesis into parameters of *P*_max_, *ϕ*, *θ*, and *R*_d_ for up and bottom layer leaves, and the plant architecture into leaf length (LL), leaf width (LW), leaf curvature (LC), and leaf angle (LA) for up and bottom layer leaves, and leaf number per plant (LN). Using the model and dissection method, we calculated the relative change of *A_c,d_* when substituting each trait of W64A by the value from A619 (Figs. 8 and 9). The relative difference of the individual traits between the 2 inbred lines was also calculated as (Trait_A619_ − Trait_W64A_)/Trait_W64A_. Leaf photosynthesis-related traits such as *P*_max_*, ϕ, θ*, and *R*_d_ were fitted from light response curves. Although leaf photosynthesis was identified as the major factor controlling the difference between W64A and A619, the impact of each trait on *A_c,d_* was unclear. Therefore, virtual canopies were created by replacing each parameter from A619 to W64A to assess the impact of each trait (Fig. 8). The relative change of these traits was different between the 2 inbred lines at different stages (Fig. 8). For example, the change of *P*_max_, *θ*, and *R*_d_ was almost the same at DAS 31, but *θ* for up layer leaves was the largest one at stage 2 (DAS 38). Leaf length of the up layer leaves, leaf number, and leaf width of the bottom layer leaves were the major contributors to the *A_c,d_* at the first stage (DAS 31) (Fig. 9F). Similarly, the major contributors were also identified at other stages (Fig. 9G to J). Notably, the contribution of a single trait to *A_c,d_* was even higher than the contribution of the complete plant architecture to *A_c,d_* in the last 3 stages (Fig. 7H to J), because different traits may have opposite effects on canopy photosynthesis (Fig. 7H to J).

From the dissection results, we can project the function of light response curve parameters and canopy architectural traits to the influence of canopy photosynthesis.

## Discussion

### Dissection of factors responsible for difference in canopy photosynthesis between cultivars

This study developed a novel 3D canopy modeling pipeline, and the contributions of plant architectural traits, leaf absorbance, and leaf photosynthetic parameters to canopy photosynthesis were dissected (Fig. 1). Quantifying the contributions of different traits is essential for studying the genetic mechanism of canopy photosynthesis. Canopy photosynthesis is a complex trait influenced by many traits, such as leaf photosynthetic efficiency, leaf absorbance, and plant architectural traits. A survey of maize cultivars has shown substantial genetic variations in maize leaf photosynthesis [22]. Leaf photosynthetic capacity can be improved by various options reviewed in Refs. [20,21,49], such as increasing leaf nitrogen content [50] and optimizing nitrogen allocation among enzymes [51,52]. In the current study, leaf photosynthetic efficiency is the primary factor controlling canopy photosynthesis (*A_c_*) with an impact of 17.5% to 29.2% to the difference of *A_c_* between the 2 varieties (Fig. 7F to J). Further analysis shows that the parameters such as *P*_max_, *ϕ*, and *θ* of the top layer and ϕ of the bottom layer significantly influenced canopy photosynthesis. While *P*_max_ determines photosynthesis at high light, *ϕ* (initial slope of the light response curve) determines the low-light CO_2_ assimilation rate, and *θ* (the convexity of light curve) influences photosynthesis under medium light levels. From the dissection analysis, we found that plant architecture determines light distribution in the canopy at different developmental stages, affecting the relative importance of *P*_max_ and initial slope of the light response curve in controlling the difference of *A_c,d_* between the 2 maize varieties. Furthermore, as chlorophyll content and antenna size determine light harvesting capacity, *ϕ* of the bottom layer leaves also influenced *A_c,d_* between the 2 maize varieties (at stages 2, 3, and 4) (Fig. 8).

Recent studies in rice show that at the bottom layer of a canopy, chlorophyll content and antenna size need to be kept or increased to improve canopy photosynthesis [53], and optimal nitrogen partitioning is required for enhancing leaf photosynthesis [54]. Nonetheless, the changes in leaf chlorophyll content did not contribute to the difference of *A_c,d_* between the 2 maize varieties (Fig. 7F to J). In contrast, previous modeling studies show that when leaf chlorophyll content decreases, more light can penetrate to the lower layer of a canopy in rice [47] and soybean [55]. The canopy photosynthesis can be increased by 3% when chlorophyll is decreased by 40% based on a modeling study [47]. In the current study, the leaf transmittance of A619 was 2% to 4% lower than W64A, while leaf reflectance was about 1% different between 2 varieties at the first 2 stages and nearly the same at other stages (Fig. 3K and L). The relatively small difference of leaf absorbance between the 2 varieties explained the little contribution of *A_c,d_* (Fig. 7F to J).

A limitation of the dissection method is that gene linkage and gene pleiotropy cause trade-offs between traits, making it challenging to decouple them. Therefore, a recently published technology to target a gene regulatory element has been proposed to address this challenge [56]. Another limitation of the current method is that it does not include total nitrogen content in the virtual canopy simulations. There are variations in photosynthetic properties for leaves at different positions of a canopy. The nitrogen content in the canopy can be used to estimate the leaf photosynthetic properties for different leaves, and hence better calculate whole canopy photosynthetic efficiency [50].

### Factors controlling canopy photosynthesis in a maize canopy

Dissection of factors that are responsible for the difference in canopy photosynthesis between cultivars can help guide combination of traits to improve canopy photosynthesis for a particular cultivar. Similar to this current study, such analysis has also been done for 2 elite rice cultivars, i.e., 9311 and HuangHuaZhan [37]. Results from such analysis can immediately be used to guide current crop breeding. In addition to this, canopy photosynthesis models can be directly used to identify factors that can be modified to gain improved canopy photosynthesis through sensitivity analysis.

Our analysis using maize canopy models here show that photosynthetic parameters, such as *P*_max_, *ϕ*, and *θ*, all show a linear relationship with canopy photosynthesis (Fig. 4), while leaf respiration shows a negative relationship with canopy photosynthesis (Fig. 4). This supports the notion that improving photosynthetic efficiency is an effective approach to improve crop yield [20]. Similarly, decreasing respiration is also another major option to improve crop yield potential [57]. The observed linear relationship between canopy photosynthesis and either *P*_max_ or *ϕ* reflects that photosynthesis of both the upper leaves and lower canopy leaves together form the total canopy photosynthesis [58]. In the field, increasing leaf photosynthesis usually led to increased biomass though the percentage of canopy photosynthesis is less than the increase in leaf photosynthesis, e.g., as in the case of elevated CO_2_ [1]. More studies are needed to understand why the percentage increase in biomass is less than the percentage increase in leaf photosynthesis in the field.

Sensitivity analysis also showed that altering leaf width, leaf length, and leaf number can similarly influence *A_c,d_* (Fig. 6). Thus, it is difficult to get a correlation between one architectural trait with biomass or crop yield because these traits co-vary with each other. Leaf angle and leaf curvature are also important traits for maize (see review by Mantilla-Perez and Fernandez [59]), especially for the planting densities [60–63]. Further improving the maize planting density is regarded as a major area of research globally [64].

### Enhanced efficiency in 3D canopy modeling: A multi-view stereo approach using the MVS64 system and further improvements

The MVS64 system developed in this study significantly improved the efficiency of constructing 3D canopy models. Compared to previous developed systems that rotate plants [38,40,65,66] or cameras [41], the MVS64 took all 64 images simultaneously. Therefore, the time required for capturing images was limited to moving pots on and off the system and waiting for leaf stabilization (approximately 30 s per pot for manual movement). Another time-consuming aspect was generating the 3D point cloud from the images, which depended on the computer's processing speed. In our case, this process took approximately 20 to 40 min per plant, but parallel computing could significantly reduce this time. The MVS64 not only increased the efficiency of imaging, but also minimized errors caused by leaf movement. This system has the potential to be used in the field crop high-throughput phenotyping [67], similar to previous studies that utilized multiple cameras for field applications [68].

Some steps in this pipeline still exhibited low throughput, such as measuring leaf chlorophyll content and leaf photosynthetic light response curves. Multispectral or hyperspectral imaging has been employed to predict leaf chlorophyll content [69] and photosynthetic parameters [70]; however, challenges exist due to leaf angle and the distance between the light source and the leaves [71]. The light response curve can be estimated from leaf chlorophyll fluorescence parameters, including quantum yield of PSII (Φ_PSII_) and electronic transport rate under varying light intensities [72]. Hyperspectral reflectance can also be employed to measured leaf photosynthetic efficiency [73]. The algorithm required to align the 3D point cloud with the 2D multispectral and fluorescence image was a major challenge for integrating all of these high-throughput technologies in 3D canopy photosynthesis modeling. The 3D canopy modeling pipeline can support not only the MVS64 system, but also other facilities capable of generating point clouds. For the purpose of reducing facility costs, other types of MVS systems developed in previous studies [38–41], as well as LiDAR and depth camera, can be utilized.

The organ segmentation is essential for the 3D canopy modeling pipeline, and the algorithms for point cloud segmentation are required for different crops. Accurate extraction of plant architectural parameters is also very important. In the current study, the maize plants have wavy leaf blade edges, which introduced error to both the manually measured and the point cloud-based calculated leaf width (Fig. 2F and J). For example, it is difficult to visually determine the maximal leaf width. In contrast, the accuracy of leaf length measurement and calculation was higher, because the leaf length is several times longer than leaf width and the relative error of the measurement was smaller.

## Conclusion

This study presents a novel pipeline that offers a method to elucidate the connection between individual phenotypes controlling the complex trait of canopy photosynthesis. Utilizing the pipeline, we observed a linear correlation between leaf photosynthetic parameters and canopy photosynthesis (*A_c,d_*) in most circumstances. On the other hand, the relationship of canopy architectural traits with *A_c,d_* was nonlinear, and the optimal values depended on the plant architecture and the growth stages. Our findings demonstrated that leaf photosynthesis was the primary determinant (17.5% to 29.2%) for the disparity in canopy photosynthesis (*A_c,d_*) between the 2 investigated maize varieties, across all growth stages. In-depth analysis revealed the contributions of maximal photosynthetic rate (*P*_max_), quantum yield (*ϕ*), and the convexity of light response curve (*θ*) for leaves at the upper and lower layers of canopies. Canopy architecture served as the secondary factor (5.3% to 6.7%), influencing the difference of *A_c,d_* between the 2 varieties at early stages, with the leaf width, leaf length, and leaf number being the major contributors. The pipeline can be used as a general strategy to support current ideotype breeding practices for enhancing crop yield and represents a new field of application for the modern high-throughput phenomics facilities.

## Data Availability

The source code used in this study is available for non-commercial use and the code can be downloaded from https://github.com/PlantSystemsBiology/3DCanopyModel. The *FastTracer* software is available from https://github.com/PlantSystemsBiology/fastTracerPublic.
