# Supplementary material for: Quantifying Contributions of Different Factors to Canopy Photosynthesis in 2 Maize Varieties: Development of a Novel 3D Canopy Modeling Pipeline
Source: Plant Phenomics. 2023 Jul 26;5:0075. doi: 10.34133/plantphenomics.0075 (PMC10371248; doi:10.34133/plantphenomics.0075)
Supplement: Supplementary 1 — Figs. S1 to S5 Tables S1 to S3 Supplementary methods [file plantphenomics.0075.f1.zip › Supplementary data 2023-7-12 clean.docx]

**Supplementary data**

**Quantifying Contributions of Different Factors to Canopy Photosynthesis in Two Maize Varieties: Development of a Novel 3D Canopy Modeling Pipeline**

Qingfeng Song^1^, Fusang Liu^1^, Hongyi Bu^2^, Xin-Guang Zhu^1^

^1^National Key Laboratory of Plant Molecular Genetics, CAS Center for Excellence in Molecular Plant Sciences, Shanghai Institute of Plant Physiology and Ecology, Chinese Academy of Sciences, Shanghai 200032, China

^2^Shanghai Institute of Technical Physics, Chinese Academy of Sciences, Shanghai, China


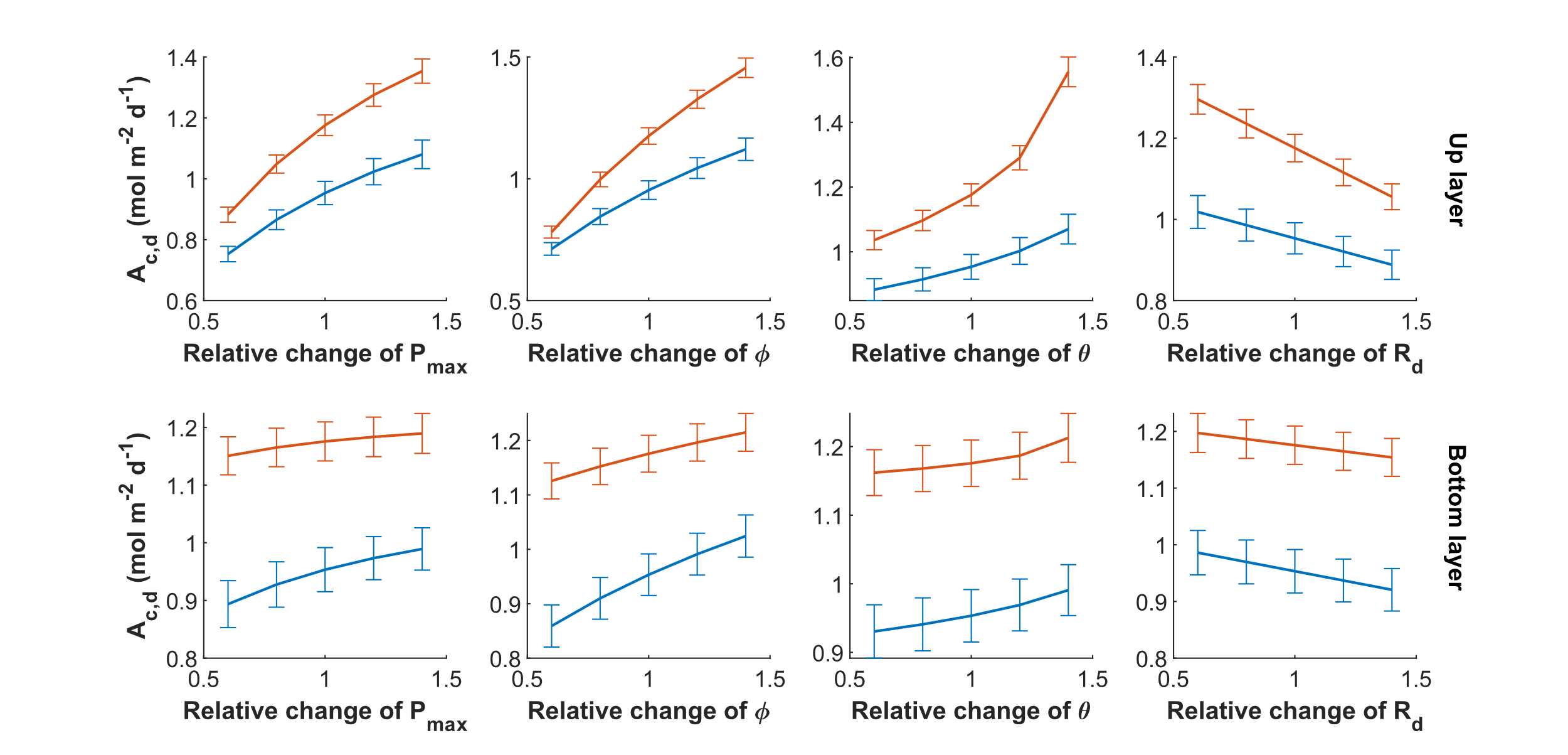


Figure S1, relationship between leaf photosynthetic parameters (up and bottom layer leaves) and canopy photosynthesis rate (A_c,d_) at the first stage (DAS 31) for the two maize inbred lines (blue line: W64A; orange line: A619) derived with the modeling pipeline. Leaf photosynthetic parameters includes the maximal photosynthesis rate *P_max_*, quantum yield *φ*, convexity of light curve *θ* and respiration rate R_d_ of up layer (a-d) and bottom layer leaves (e-h). Data used for building models were measured data on the 31^st^ day after sowing. Data were shown as mean±sd (n=5 repeats of model simulation).


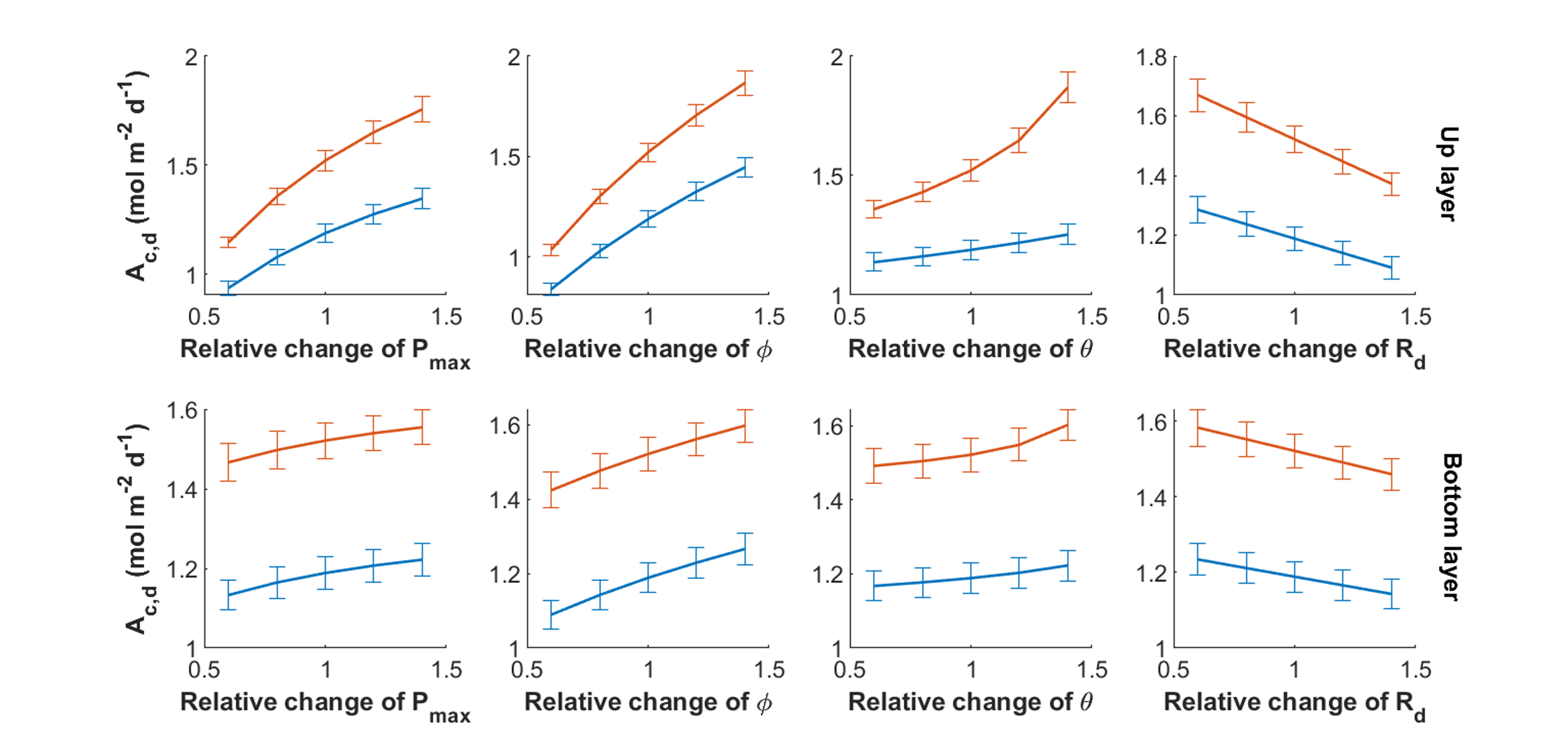


Figure S2, relationship between leaf photosynthetic parameters (up and bottom layer leaves) and canopy photosynthesis rate (A_c,d_) at the third stage (DAS 45) for the two maize inbred lines (blue line: W64A; orange line: A619) derived with the modeling pipeline. Leaf photosynthetic parameters includes the maximal photosynthesis rate *P_max_*, quantum yield *φ*, convexity of light curve *θ* and respiration rate R_d_ of up layer (a-d) and bottom layer leaves (e-h). Data used for building models were measured data on the 45^th^ day after sowing. Data were shown as mean±sd (n=5 repeats of model simulation).


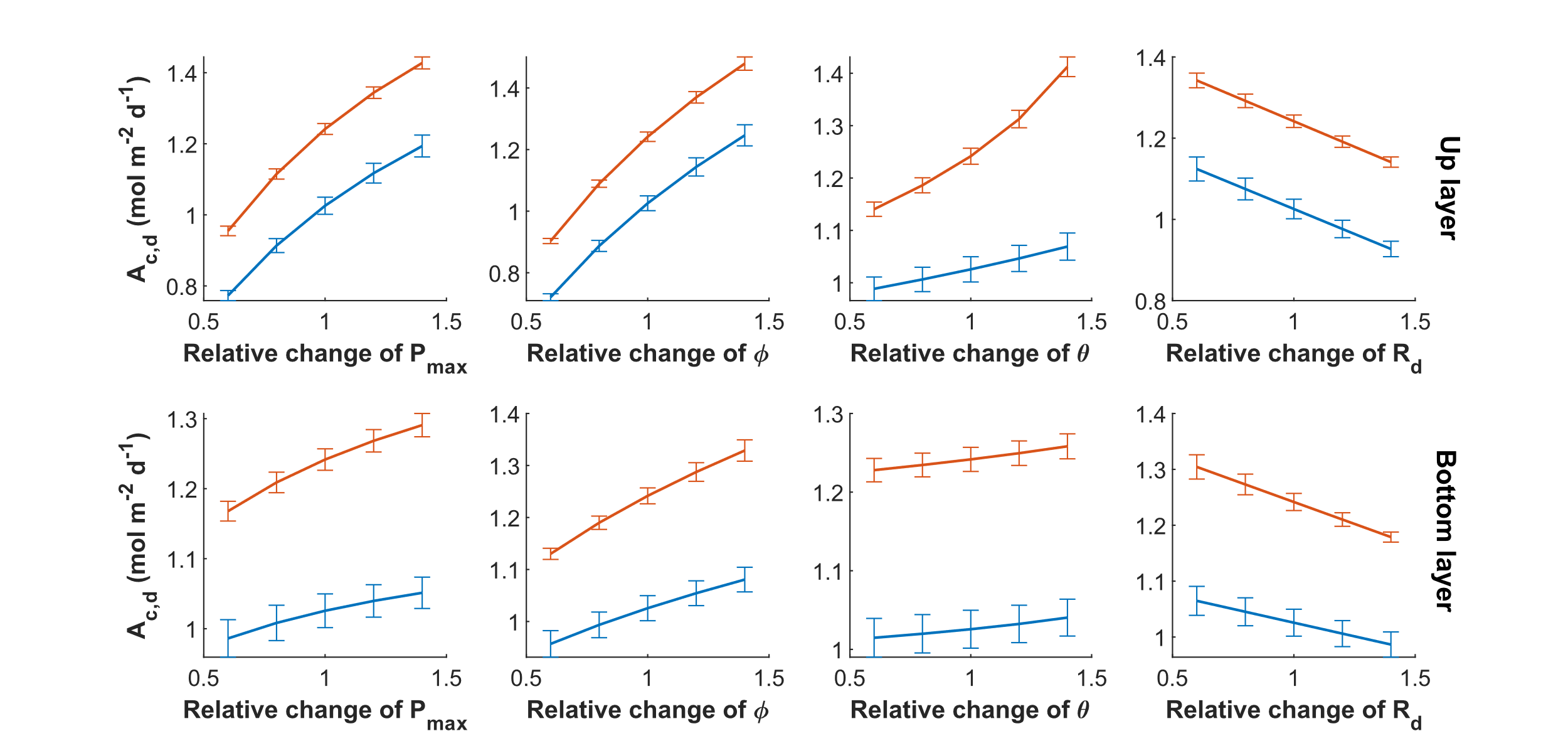


Figure S3, relationship between leaf photosynthetic parameters (up and bottom layer leaves) and canopy photosynthesis rate (A_c_) at the fourth stage (DAS 52) for the two maize inbred lines (blue line: W64A; orange line: A619) derived with the modeling pipeline. Leaf photosynthetic parameters includes the maximal photosynthesis rate *P_max_*, quantum yield *φ*, convexity of light curve *θ* and respiration rate R_d_ of up layer (a-d) and bottom layer leaves (e-h). Data used for building models were measured data on the 52^nd^ day after sowing. Data were shown as mean±sd (n=5 repeats of model simulation).


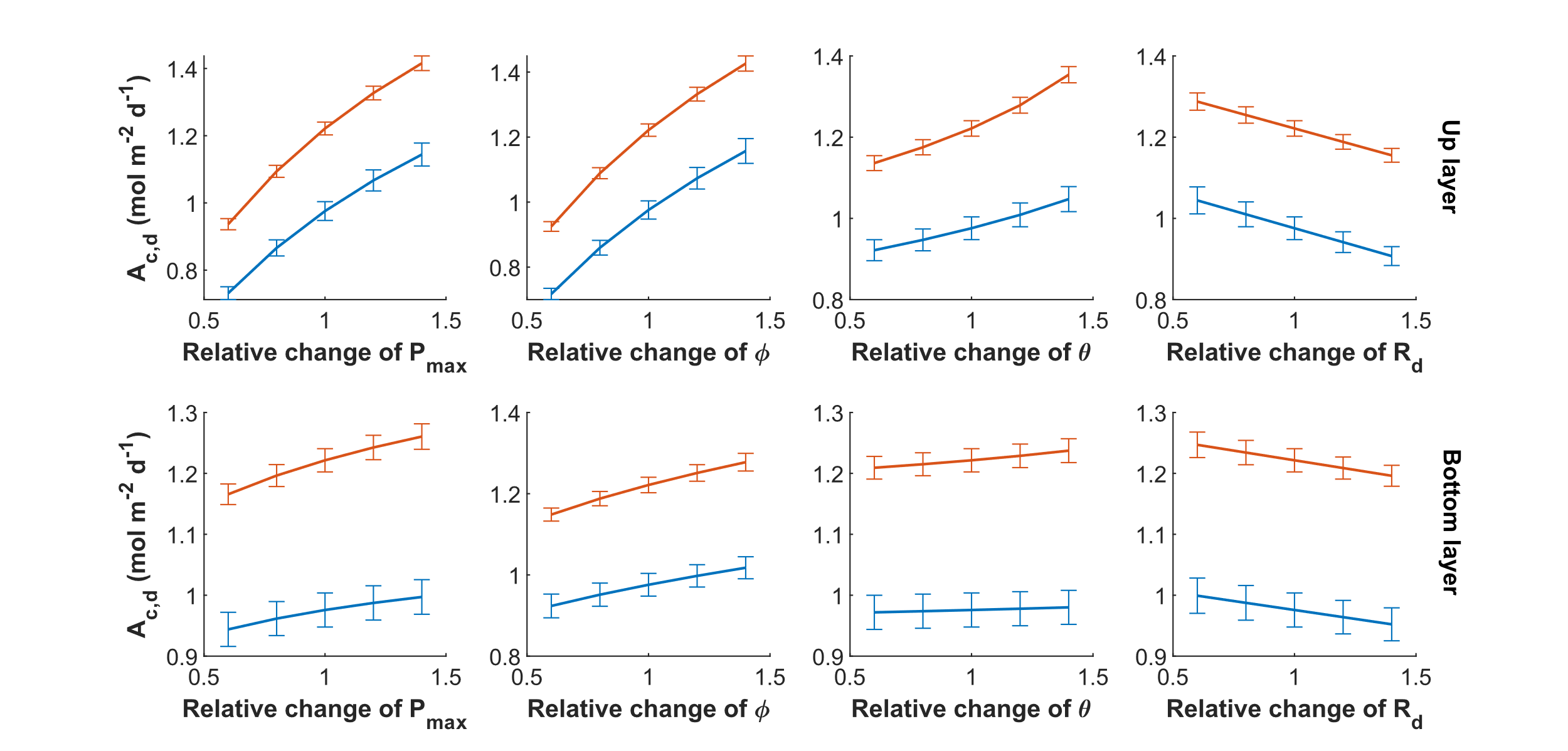


Figure S4, relationship between leaf photosynthetic parameters (up and bottom layer leaves) and canopy photosynthesis rate (A_c_) at the fifth stage (DAS 59) for the two maize inbred lines (blue line: W64A; orange line: A619) derived with the modeling pipeline. Leaf photosynthetic parameters includes the maximal photosynthesis rate *P_max_*, quantum yield *φ*, convexity of light curve *θ* and respiration rate R_d_ of up layer (a-d) and bottom layer leaves (e-h). Data used for building models were measured data on the 59^th^ day after sowing. Data were shown as mean±sd (n=5 repeats of model simulation).


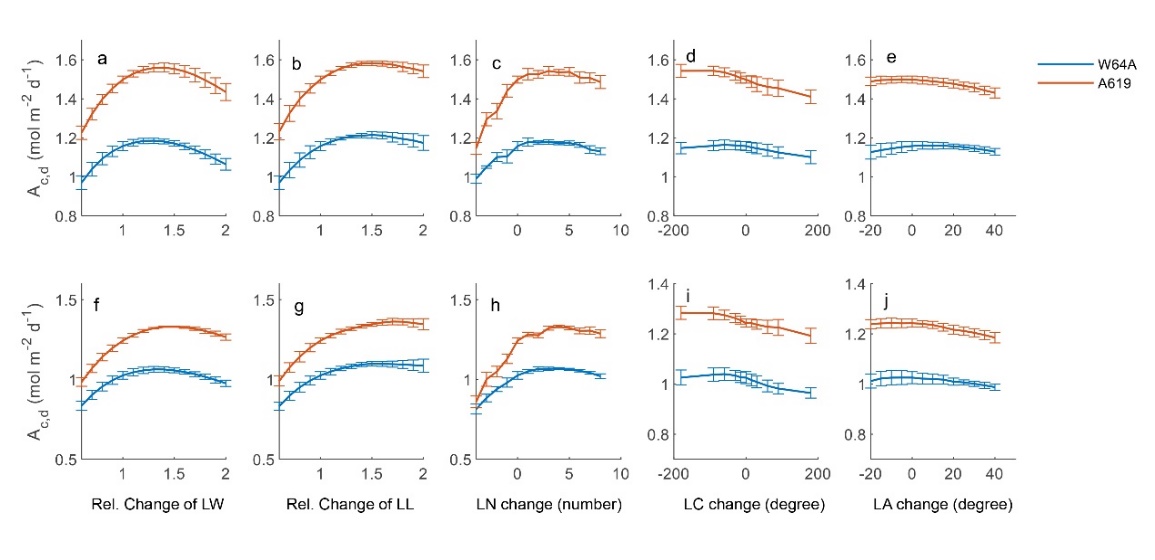


Figure S5, non-linear relationship between plant architectural traits and daily canopy photosynthesis (A_c,d_) at different stages: DAS 45 (a-e) and DAS 52 (f-j) for the two maize W64A (blue line) and A619 (orange line) calculated with the modeling pipeline. Plant architectural parameters are LW: leaf width; LL: leaf length; LN: leaf number; LC: leaf curvature and LA: leaf angle. The relative change of traits (for LW and LL) was the multiplied factor based on the original value of cultivar W64A and A619. The LN change number was the leaf number increased or decreased. The LC and LA change degree was the degree increased or decreased based on the original plant. Data were shown as mean±sd (n=5 repeats of model simulation).

Table S1, The parameters of fitting light response curves with the non-rectangular hyperbola model. Data are shown as mean ± sd (n=5). The parameters include ***P_max_*** (unit: μmol m^-2^ s^-1^), *Φ* (unit: μmol CO_2_ μmol^-1^ photon), ***θ*** (dimensionless) and ***R_d_*** (unit: μmol m^-2^ s^-1^) at five stages on the 31^st^, 38^th^, 45^th^, 52^nd^, 59^th^ days after sowing (DAS). The upper layer leaves and lower layer leaves are analyzed and presented separately.

| **Parameter** | **Stage**  (DAS) | **Upper layer leaf** | | **Lower layer leaf** | |
| --- | --- | --- | --- | --- | --- |
|  |  | **W64** | **A619** | **W64** | **A619** |
| ***P_max_*** | 31 | 56.71±9.41 | 66.60±6.06 | - | - |
|  | 38 | 60.42±11.77 | 60.29±2.48 | 43.77±3.45 | 44.07±2.92 |
|  | 45 | 55.70±9.55 | 59.57±5.15 | 36.05±4.72 | 47.42±8.95 |
|  | 52 | 46.32±11.28 | 48.00±3.31 | 28.90±2.75 | 35.24±6.26 |
|  | 59 | 35.65±2.99 | 40.97±5.92 | 26.65±3.90 | 27.81±4.35 |
| ***Φ*** | 31 | 0.061±0.007 | 0.067±0.002 | - | - |
|  | 38 | 0.060±0.005 | 0.070±0.005 | 0.052±0.009 | 0.064±0.011 |
|  | 45 | 0.055±0.003 | 0.066±0.003 | 0.044±0.006 | 0.066±0.014 |
|  | 52 | 0.060±0.007 | 0.068±0.005 | 0.051±0.010 | 0.065±0.013 |
|  | 59 | 0.057±0.007 | 0.072±0.005 | 0.066±0.004 | 0.075±0.005 |
| ***θ*** | 31 | 0.61±0.15 | 0.72±0.13 | - | - |
|  | 38 | 0.40±0.33 | 0.69±0.16 | 0.60±0.14 | 0.77±0.18 |
|  | 45 | 0.44±0.31 | 0.64±0.06 | 0.69±0.05 | 0.39±0.19 |
|  | 52 | 0.32±0.30 | 0.62±0.14 | 0.51±0.21 | 0.39±0.23 |
|  | 59 | 0.47±0.30 | 0.59±0.20 | 0.27±0.19 | 0.48±0.24 |
| ***R_d_*** | 31 | 4.33±0.83 | 5.14±0.51 | - | - |
|  | 38 | 3.63±0.49 | 4.46±0.50 | 2.89±0.46 | 3.74±0.54 |
|  | 45 | 3.26±0.32 | 3.70±0.61 | 2.39±0.43 | 2.75±0.52 |
|  | 52 | 3.32±0.75 | 3.76±0.65 | 2.09±0.42 | 2.75±1.02 |
|  | 59 | 2.26±0.25 | 2.41±0.28 | 1.56±0.20 | 1.64±0.20 |

Table S2, The *p* values of Students’ t test for the leaf photosynthesis rate between W64A and A619 under different PPFDs (unit: μmol m^-2^ s^-1^) at different stages (31, 38, 45, 52 and 59 days after sowing, DAS). The up layer and bottom layer leaves were measured and analyzed, respectively.

| PPFD | Up layer | | | | |  | Bottom layer | | | |
| --- | --- | --- | --- | --- | --- | --- | --- | --- | --- | --- |
|  | 31 DAS | 38 DAS | 45 DAS | 52 DAS | 59 DAS |  | 38 DAS | 45 DAS | 52 DAS | 59 DAS |
| 2000 | **0.0006** | **0.0001** | **0.0017** | **0.0180** | **0.0069** |  | 0.0547 | 0.1438 | 0.1416 | 0.4007 |
| 1600 | **0.0006** | **0.0003** | **0.0018** | **0.0123** | **0.0036** |  | **0.0077** | 0.1656 | 0.1362 | 0.2258 |
| 1200 | **0.0006** | **0.0006** | **0.0009** | **0.0060** | **0.0023** |  | **0.0024** | 0.1774 | 0.1462 | 0.1436 |
| 1000 | **0.0003** | **0.0006** | **0.0002** | **0.0014** | **0.0016** |  | **0.0014** | 0.2076 | 0.1464 | 0.1125 |
| 800 | **0.0002** | **0.0003** | **0.0002** | **0.0002** | **0.0005** |  | **0.0007** | 0.1883 | 0.2007 | 0.0963 |
| 600 | **0.0041** | **0.0002** | **0.0004** | **0.0001** | **0.0001** |  | **0.0007** | 0.0676 | 0.1358 | 0.0696 |
| 400 | **0.0356** | **0.0009** | **0.0004** | **0.0019** | **0.0001** |  | **0.0003** | **0.0304** | 0.1158 | **0.0191** |
| 300 | 0.0759 | **0.0021** | **0.0035** | **0.0040** | **0.0003** |  | **0.0045** | **0.0328** | **0.0360** | **0.0057** |
| 200 | 0.2611 | **0.0207** | **0.0003** | **0.0061** | **0.0007** |  | **0.0147** | **0.0365** | 0.0551 | **0.0013** |
| 150 | 0.5725 | 0.0868 | **0.0011** | **0.0277** | **0.0006** |  | **0.0271** | **0.0419** | 0.0861 | **0.0021** |
| 100 | 0.8534 | 0.4984 | **0.0339** | **0.0157** | **0.0005** |  | 0.4270 | 0.0624 | 0.1384 | **0.0031** |
| 50 | 0.0651 | 0.1126 | 0.6485 | 0.8074 | **0.0026** |  | 0.0669 | 0.1932 | 0.8711 | 0.0955 |
| 0 | 0.1004 | **0.0283** | 0.1914 | 0.3536 | 0.3874 |  | **0.0272** | 0.2966 | 0.2688 | 0.5098 |

Table S3, Comparison of different platforms for acquiring point clouds of maize.

| Technology | Sensor | Plant | Time of scanning or taking images (plant^-1^) | Literature |
| --- | --- | --- | --- | --- |
| LiDAR | LiDAR, fixed | Rotation | 120 s | Thapa et al, 2018 |
|  | LiDAR, multiple station | Fixed | 33 mins | Current study |
| SFM-MVS | Manually, one camera | Fixed | 5-10min | Duan et al., 2016 |
|  | Manually, one camera | Fixed | 1.5-4 mins | Wang et al., 2019 |
|  | Two fixed cameras | Rotation | 3 mins (72 images) or  30 mins (360 images) | Nguyen et al., 2016 |
|  | 1-3 cameras, rotation | Fixed | 60 – 120 s | Wu et al, 2020 |
|  | 64 cameras, fixed | Fixed | **< 0.1 s** | Current study |

**SUPPLEMENTARY METHODS:**

**Point cloud reconstruction.**

Images from the phenotyping platform are used to generate dense point clouds by the software Agisoft Metashape Professional Edition (Agisoft LLC, St. Petersburg, Russia; version 1.6.1), which is based on the structure from motion (SFM) algorithm with multi-view stereo (MVS) systems (Smith *et al.*, 2016, Webster *et al.*, 2018). The positions of all cameras are fixed on the mechanical structure of the platform, and the settings of the cameras are also fixed during the phenotyping for one stage, so these data are pre-calibrated and recorded in a configuration file, which are imported to the software for the calculation of dense point clouds. High-quality dense point clouds are constructed with the function of “high quality” from the software. The process of point cloud calculation includes three steps. First, key points corresponding to specific features of images are detected by a SIFT (Scale Invariant Feature Transform) algorithm (Lowe, 2004). Second, the sparse point clouds are generated using the key points based on the SFM algorithm (Jaud *et al.*, 2016). Third, the MVS algorithm uses sparse point clouds to construct dense point clouds (Weiss & Baret, 2017). The point clouds data include the information of X-Y-Z coordinates, color (RGB) and normal vector (of the object surface, such as a leaf or a stem) of the points.

**Plant organ segmentation and architectural parameter extraction**

Support vector machine (SVM) models are trained to classify the original point clouds of the plant (**Fig. 1c**) as target (plant and soil, **Fig. 1c**) and background (white noise) points based on color information. MATLAB (R2020b, MathWorks, USA) was used in the following analysis. The soil plane is extracted by the function *pcfitplane*, which uses algorithm of M-estimator sample Consensus to find a plane within point clouds (Torr & Zisserman, 2000). With the algorithm, a plane is fitted to a point cloud with allowable distance between inlier points and the plane not larger than 5 cm. The vegetation point clouds are then denoised with the *pcsegdist* and *pcdenoise* function. The larger outliers was removed using *pcsegdist* with a Euclidean distance threshold 5 mm, and then the small outliers is removed using *pcdenoise* with a threshold of standard deviation 0.3 away from the averaged distance of between each point and its nearest 50 neighbors (**Fig. 2c**).

The method of plant organ segmentation developed by (Liu *et al.*, 2021b) is first used to separate every leaf from the stem. The method combines skeleton extraction algorithm and region growing algorithm and is demonstrated to be effective for maize (Liu *et al.*, 2021a). The algorithm includes three major parts, (1) extracting leaf skeleton, (2) classifying point cloud to clusters and (3) merging unknown clusters. After separating all leaves from the stem, extraction of leaf architectural traits is applied with the method used in (Liu et al., 2021b). The leaf architectural traits include leaf length, leaf width, leaf base height and leaf area. The leaf area is calculated based on the length and width of a leaf with an empirical model (Dornbusch *et al.*, 2011). The extracted traits are evaluated according to the measured data by calculating R^2^ and RMSE.

**Building 3D canopy photosynthesis model based on point cloud data**

Point clouds of single plant are converted into mesh models and used for constructing virtual canopies (**Fig 1d, e**). The Crust method (Amenta *et al.*, 1998) was used for triangulation, then abnormal facets (or triangles) are filtered with a statistic method used in previous study (Liu et al., 2021a). Each canopy model is built with four different individual plants and the data of the four plants are used repeatedly. One canopy model includes 4 rows with 55 cm distance between rows and 13 plants per row with 15 cm distance between plants. The center area of 110 cm * 75 cm (2 rows * 5 plants/row) are used for ray tracing simulation and canopy photosynthesis calculation to avoid boundary effect (**Fig. 1f**).

The light distribution in canopy is simulated with ray tracing algorithm using software *FastTracer* (Song *et al.*, 2013). Data points represent photosynthetic photon flux density (PPFD) absorbed by triangles of leaves in a canopy (**Fig. 1g**). The meta information including date and location are used as input to the software. The *FastTracer* software is available from Github (<https://github.com/songqingfeng/fastTracerPublic>).

To build the canopy photosynthesis model, the classic non-rectangular hyperbola leaf photosynthesis model (**Eqn. S1**) (Thornley, 2002) is used to calculate photosynthetic CO_2_ assimilation rate for every triangle of individual leaves in a canopy model according to the absorbed PAR by each triangle (**Fig. 1g,h**). *A* is leaf photosynthesis rate, *P_max_* is leaf photosynthetic CO_2_ assimilation rate under saturate light. $\phi$ is quantum yield of CO_2_ assimilation. $\theta$ is the curve convexity, which describes the sharpness of the transition in the light response curve.

$A=\frac{\phi I+P_{max}-\sqrt{{(\phi I+P_{max})}^{2}-4\theta\phi IP_{max}}}{2\theta}$ (S1)

The canopy photosynthesis rate equals the sum of all leaf photosynthesis rate multiplied by leaf area. Diurnal canopy photosynthesis rate is calculated based on the simulated light environments on an hourly interval (**Fig. 1i**).

**Setup of LiDAR device for validation of point cloud accuracy**

To verify the accuracy of 3D point clouds acquired from the multi-view stereo system, we used LiDAR (FARO S70 series) to scan plants for point clouds (**Fig. 2a**). To obtain highly accurate point clouds, the LiDAR was used with the setting of high accuracy. For each plant, LiDAR-scanning data from three or more angles (stations) were needed to construct a complete 3D point cloud with LiDAR, which means the LiDAR device scan the plant from different angles. To reduce the time for scanning, we set up the LiDAR so that we can scan multiple plants together, such as scanning three plants using four stations, with scanning orientation from 0 to 120 degrees for the surrounding stations and orientation from 0 to 360 degrees for the center station (**Fig. 2b**). Point cloud data from all the stations were constructed by the positioning balls and separated into individual plants with the software of SCENE 2019 (together with the LiDAR device).

**Manual measurement of plant architectural traits for evaluating point cloud segmentation algorithms**

We compared measurements from the phenotyping platform and those from traditional manual measurements. Plant architectural traits, *i.e.* leaf base height, leaf length, leaf width for all green leaves of a plant were carefully manually measured without destructing plants. The leaf base height is the distance between the leaf-stem intersection point and the soil surface. Leaf length is the length of a stretched leaf. Leaf width was measured at different positions of a leaf, *i.e.* leaf base, 1/4, 1/2 and 3/4 of leaf length from the leaf base, and the maximal width was used for the analysis.

**Reference**

Amenta, N., Bern, M. and Kamvysselis, M. (1998) A new voronoi-based surface reconstruction algorithm. *Proceedings of the 25th Annual Conference on Computer Graphics and Interactive Techniques, SIGGRAPH 1998***,** 415-422.

Dornbusch, T., Watt, J., Baccar, R., Fournier, C. and Andrieu, B. (2011) A comparative analysis of leaf shape of wheat, barley and maize using an empirical shape model. *Annals of Botany,* **107,** 865-873.

Jaud, M., Passot, S., Le Bivic, R., Delacourt, C., Grandjean, P. and Le Dantec, N. (2016) Assessing the accuracy of high resolution digital surface models computed by PhotoScan® and MicMac® in sub-optimal survey conditions. *Remote Sensing,* **8,** 465.

Liu, F., Hu, P., Zheng, B., Duan, T., Zhu, B. and Guo, Y. (2021a) A field-based high-throughput method for acquiring canopy architecture using unmanned aerial vehicle images. *Agricultural and Forest Meteorology,* **296,** 108231.

Liu, F., Song, Q., Zhao, J., Mao, L., Bu, H., Hu, Y.*, et al.* (2021b) Canopy occupation volume as an indicator of canopy photosynthetic capacity. *New Phytologist,* **232,** 941-956.

Lowe, D. G. (2004) Distinctive image features from scale-invariant keypoints. *International Journal of Computer Vision,* **60,** 91-110.

Smith, M. W., Carrivick, J. L. and Quincey, D. J. (2016) Structure from motion photogrammetry in physical geography. *Progress in Physical Geography,* **40,** 247-275.

Song, Q., Zhang, G. and Zhu, X.-G. (2013) Optimal crop canopy architecture to maximise canopy photosynthetic CO2 uptake under elevated CO2- a theoretical study using a mechanistic model of canopy photosynthesis. *Functional Plant Biology,* **40,** 109-124.

Thornley, J. H. M. (2002) Instantaneous canopy photosynthesis: Analytical expressions for sun and shade leaves based on exponential light decay down the canopy and an acclimated non-rectangular hyperbola for leaf photosynthesis. *Annals of Botany,* **89,** 451-458.

Torr, P. H. S. and Zisserman, A. (2000) MLESAC: A new robust estimator with application to estimating image geometry. *Computer Vision and Image Understanding,* **78,** 138-156.

Webster, C., Westoby, M., Rutter, N. and Jonas, T. (2018) Three-dimensional thermal characterization of forest canopies using UAV photogrammetry. *Remote Sensing of Environment,* **209,** 835-847.

Weiss, M. and Baret, F. (2017) Using 3D point clouds derived from UAV RGB imagery to describe vineyard 3D macro-structure. *Remote Sensing,* **9,** 111.
